# Establishing an Old Age Liaison Psychiatry Network

**DOI:** 10.1192/bjo.2022.132

**Published:** 2022-06-20

**Authors:** Josie Jenkinson, Mani Krishnan, Annabel Price, Liz Sampson

**Affiliations:** 1 Surrey and Borders Partnership NHS Foundation Trust, Chertsey, United Kingdom; 2 Tees Esk and Wear Valleys NHS Foundation Trust, Stockton on Tees, United Kingdom; 3 Cambridgeshire and Peterborough NHS Foundation Trust, Cambridge, United Kingdom; 4 East London NHS Foundation Trust, London, United Kingdom

## Abstract

**Aims:**

Our aim is to establish a network for clinicians working in or with an interest in the growing specialty of old age liaison psychiatry to provide peer support (inclusive of disciplines and geography) and access to CPD opportunities, to raise the profile of this subspecialty and enable it to continue to develop, to facilitate collaboration and integration with related disciplines and pathways and to strengthen the voice of clinicians in lobbying for improvements in mental health services for older people in the general hospital.

**Methods:**

Old Age Liaison Psychiatry is a growing subspecialty in the UK and nationally, following widespread investment in development of liaison services in line with Department of Health strategy. With this expansion comes an increasing need for continuous professional development, networking and collaboration opportunities in order to nurture the specialty and those working in it.

**Results:**

Over 100 people registered for the initial webinar, and many more have watched the recording. Since the webinar the network has grown to 350 members. The webinars were received very positively, with many suggestions made for topics to be covered at future events.

**Conclusion:**

The network has been established successfully and founders are now planning future events with the support of the Royal College of Psychiatrists, including a half day learning event in late 2022.

